# The Non-inferiority of Telemedicine in Pre-anesthesia Clinic at a Tertiary Cancer Care Center: A Randomized Study

**DOI:** 10.7759/cureus.78596

**Published:** 2025-02-05

**Authors:** Prateek Maurya, Raghav Gupta, Seema Mishra, Sachidanand Bharati, Nishkarsh Gupta, Rakesh Garg, Vinod Kumar, Sunil Kumar, Sushma Bhatnagar

**Affiliations:** 1 Department of Onco-Anesthesia and Palliative Medicine, Dr. B. R. Ambedkar Institute Rotary Cancer Hospital, All India Institute of Medical Sciences, New Delhi, New Delhi, IND; 2 Department of Surgical Oncology, Dr. B. R. Ambedkar Institute Rotary Cancer Hospital, All India Institute of Medical Sciences, New Delhi, New Delhi, IND

**Keywords:** cancer care, non-inferiority, patient satisfaction, pre-anesthesia consultation, telemedicine

## Abstract

Background

With the increasing demand for cancer care and advancements in digital health, telemedicine has emerged as a promising tool to enhance healthcare accessibility and efficiency. Its application in pre-anesthesia consultations (PAC) has the potential to address logistical challenges while maintaining clinical quality.

Purpose

This study assesses whether telemedicine is a non-inferior alternative to traditional face-to-face pre-anesthesia consultations (PAC) for cancer surgery patients at a tertiary care center and evaluates discrepancies between PAC findings and pre-surgical assessments.

Methods

This prospective, randomized non-inferiority study included patients aged 20 years or older with access to internet and video calling. The participants were randomly allocated to either a telemedicine PAC or a face-to-face PAC group in a 1:1 ratio. An eight-item questionnaire on a 10-point scale was used to measure patient satisfaction after the consultation, while a 12-item questionnaire on the same scale assessed anesthesiologists’ satisfaction. We also compared airway examination findings between the PAC and pre-surgical assessments to detect any discrepancies.

Results

Of 120 patients assessed, 100 met the inclusion criteria and were randomized (50 per group). Baseline characteristics were similar across groups. Physician satisfaction scores were comparable between telemedicine and face-to-face consultations (7.18 versus 7.58, p=0.113), as were patient satisfaction scores (6.94 versus 7.26, p=0.251). Airway examination findings were consistent, with no significant discrepancies.

Conclusion

Telemedicine was found to be non-inferior to face-to-face consultations for pre-anesthesia assessments in terms of patient and physician satisfaction, as well as clinical accuracy. These findings support the integration of telemedicine into routine pre-anesthesia practice, particularly in high-demand settings such as cancer care. Further research is warranted to evaluate broader clinical outcomes and cost-effectiveness.

## Introduction

Telemedicine, meaning “healing at a distance,” was introduced to the world in the 1970s [1]. According to the World Health Organization (WHO), telemedicine is defined as “the delivery of healthcare services, where distance is a critical factor, by all healthcare professionals using information and communication technologies for the exchange of valid information for the diagnosis, treatment, and prevention of disease and injuries; research and evaluation; and the continuing education of healthcare providers” [2]. The potential advantages of telemedicine are numerous, including improved access to healthcare, reduced travel time for patients and doctors, and enhanced communication between peripheral and tertiary care centers [3-6]. However, despite its promise, telemedicine faces significant challenges. These include a lack of robust evidence regarding its clinical efficacy and cost-effectiveness, as well as concerns that it might threaten the traditional roles of healthcare workers. There are also feelings of depersonalization associated with telemedicine, and its broader adoption is often hindered by legal and bureaucratic obstacles [7-9].

Recently, the use of telemedicine for pre-anesthesia consultations (PAC) has gained increasing attention, particularly in high-demand settings such as oncology care. However, existing literature on telemedicine PAC is limited, and most studies to date have involved small sample sizes, limiting their generalizability. As healthcare systems face growing demands on time and resources, in-person consultations may become increasingly burdensome and costly. Telemedicine could serve as a valuable alternative, providing efficient and effective consultations while minimizing resource use.

The current study aims to evaluate the feasibility of using telemedicine for pre-anesthesia consultations in a tertiary cancer care center.

Unlike previous studies, we designed this trial as a non-inferiority study to determine whether telemedicine can deliver comparable outcomes to traditional face-to-face consultations [10,11]. Our primary objective was to assess patient and physician satisfaction with telemedicine consultations. Additionally, we sought to determine if telemedicine can provide accurate clinical evaluations by comparing pre-anesthesia findings with pre-surgical assessments, specifically examining critical indicators such as airway evaluation. This comparison is essential to ensure that telemedicine not only meets satisfaction standards but also matches the clinical accuracy of in-person assessments. By developing a tele-anesthesia platform, we aimed to improve communication with patients, provide preoperative counseling, and address clinical needs such as the management of comorbid conditions, airway examination, and the delivery of anesthesia-related instructions.

This study seeks to evaluate telemedicine as a non-inferior and practical alternative for pre-anesthesia consultations in high-demand settings such as cancer care, with reduced travel, minimized waiting times, and enhanced patient convenience as possible advantages.

## Materials and methods

This prospective, randomized non-inferiority study was conducted at the Department of Onco-Anesthesia and Palliative Medicine of All India Institute of Medical Sciences, New Delhi. The study was conducted in line with the principles of the Declaration of Helsinki. The study was approved by the Institutional Ethics Committee of All India Institute of Medical Sciences, New Delhi, (IEC-617/03.07.2020) and registered at the Clinical Trials Registry of India (CTRI/2020/08/027004). Written informed consent was obtained from all individual participants included in the study, and consent to publish was also obtained from each participant.

Study participants

A total of 100 patients aged 20 years or older, scheduled for elective cancer surgery at our institution between 10/08/2020 and 09/08/2021, with internet access and video calling capabilities, were eligible for inclusion. Patients were excluded if they had acute infections requiring auscultation, were scheduled for high-risk procedures, had impaired cognitive function, or refused to provide informed consent.

Randomization and blinding

Patients meeting the inclusion criteria were randomly assigned to either a telemedicine PAC group (Group A) or a face-to-face PAC group (Group B) using block randomization with a block size of 4, generated by computer software. The randomization sequence was concealed using opaque, sequentially numbered sealed envelopes, which were opened by the attending anesthesiologist immediately before the PAC. Due to the nature of the interventions, blinding was not possible for the anesthesiologists or patients, but data analysis was performed by a blinded statistician.

Group A (telemedicine PAC)

Patients in this group received a video call for their pre-anesthesia consultation during regular outpatient department (OPD) hours. A standardized PAC assessment form was used to record detailed medical histories. The airway examination and general examination were conducted through video, with instructions to the patient (e.g., “open your mouth” and “show your nails”).

Preoperative counseling was provided, including fasting instructions, medication adjustments, and the optimization of comorbid conditions. Any additional consultations with specialists (e.g., cardiology or endocrinology) were recommended based on clinical history. Blood tests and radiology results were retrieved from the hospital’s electronic health database. Upon admission for surgery, patients were reassessed, and any discrepancies between the initial telemedicine PAC findings and pre-surgical assessments were noted.

Group B (face-to-face PAC)

Patients in this group underwent conventional, in-person pre-anesthesia consultations. Similar to Group A, medical histories were recorded, and airway and general examinations were performed. Preoperative counseling and necessary medical optimization were provided.

Blood and radiology results were reviewed from the hospital’s electronic health records, and patients were reassessed upon admission for surgery.

Questionnaires

After the pre-anesthesia consultation, patients in both groups completed an eight-item questionnaire on a 10-point scale assessing their satisfaction with the consultation. Similarly, anesthesiologists filled out a 12-item questionnaire assessing their satisfaction with the thoroughness and clarity of the preoperative evaluation. The validation of the questionnaire was conducted systematically to ensure its robustness and appropriateness. Specifically, face validity was inherently assessed as a part of the expert review process. The questionnaire was designed and shared with a panel of five experts, who evaluated each question based on criteria such as relevance, simplicity, clarity, and ambiguity. This evaluation ensured that the questions appeared appropriate and meaningful for the intended purpose, thereby confirming their adequacy in capturing the intended constructs.

The primary focus of validation was on content validity, which was quantified using the content validity index. The expert panel ensured that the questions were aligned with relevant aspects such as satisfaction levels, consultation quality, and clarity of communication. Any ambiguities raised during the evaluation process were resolved through consensus, resulting in complete agreement among all five experts.

Outcomes

The primary outcome was patient and physician satisfaction, measured using the questionnaires. Satisfaction was assessed across various factors, such as comfort during the conversation, comprehension, and thoroughness of the evaluation.

A secondary outcome was the comparison between the clinical findings from the pre-anesthesia consultation (telemedicine or face-to-face) and the pre-surgical assessment, particularly focusing on airway examination parameters. Discrepancies between the initial PAC findings and the pre-surgical assessments were documented and analyzed.

Statistical analysis

A pilot study of 60 patients was conducted to determine the sample size. Based on the results, with a power of 80% and a type I error of 5%, the required sample size was calculated as 43 patients per group. To account for potential dropouts, we included 50 patients in each group.

Quantitative data were presented as means and standard deviations, while qualitative data were presented as frequencies and percentages. Between-group comparisons for quantitative data were performed using either the t-test or the Wilcoxon rank sum test, depending on data distribution. Categorical variables were compared using the chi-square test or Fisher’s exact test. A non-inferiority margin was set for the satisfaction scores, and a two-sided 95% confidence interval was calculated to assess non-inferiority. The data were analyzed using the Stata software (version 17.0) (StataCorp LLC, College Station, TX).

## Results

**Figure 1:**
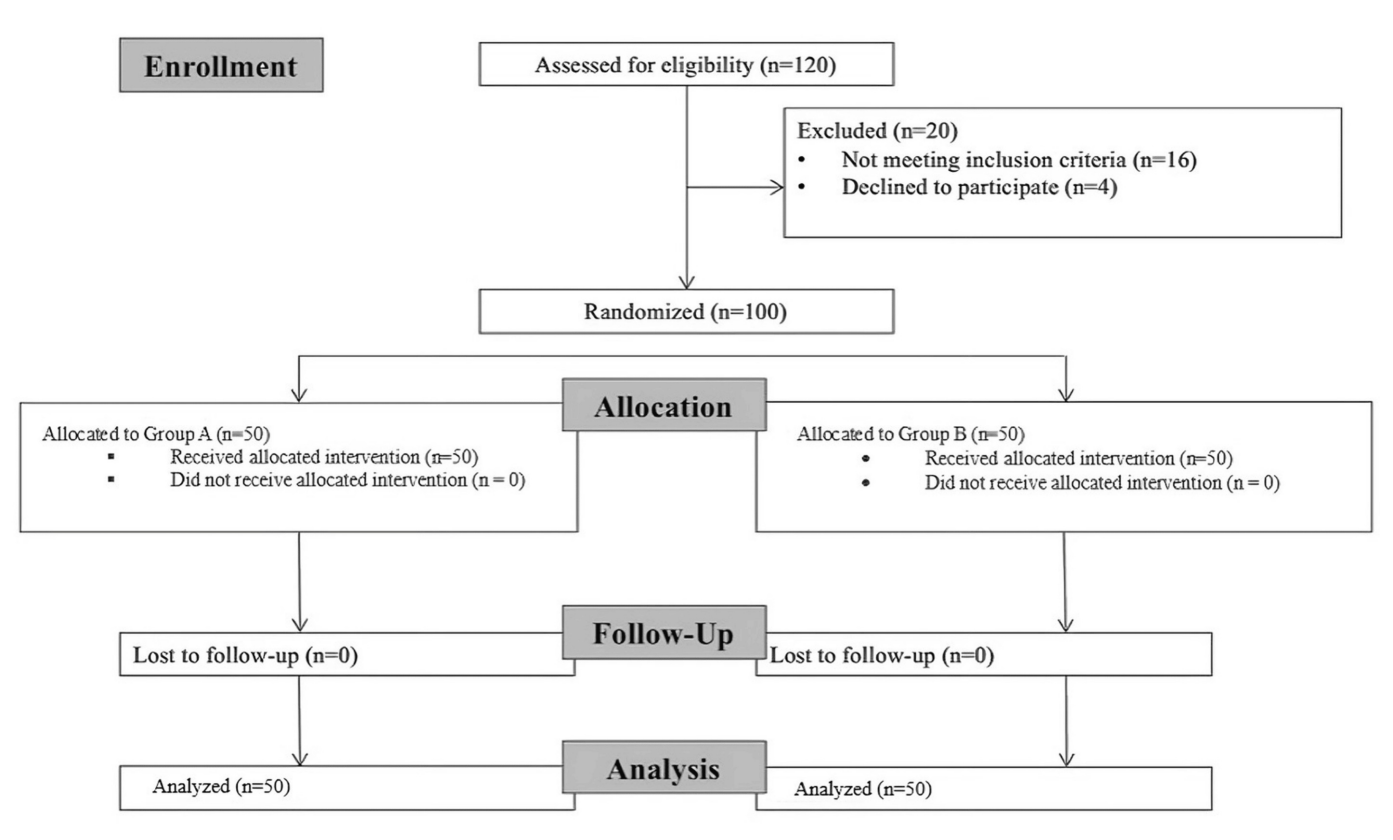
Consolidated Standards of Reporting Trials (CONSORT) flow diagram

Out of 120 patients assessed for eligibility, 20 were excluded based on the exclusion criteria. The remaining 100 patients who met the inclusion criteria were randomized equally into two groups: face-to-face PAC (Group B) and telemedicine PAC (Group A), with 50 patients in each group. There were no dropouts during the study, and the final analysis included all 100 participants. A Consolidated Standards of Reporting Trials (CONSORT) flow diagram depicting the trial is shown in Figure 1.

Baseline characteristics

As shown in Table 1, there were no statistically significant differences between the two groups in terms of age, sex distribution, height, or weight. Additionally, comorbidities such as diabetes, hypertension, and hypothyroidism were similarly distributed between the two groups, indicating comparable patient profiles. The surgical distribution across the groups also showed no significant variation, with similar proportions of patients undergoing breast, gastrointestinal, gynecological, head and neck, and other surgeries.

**Table 1:** Comparison of demographic and clinical parameters between Group A and Group B Values are presented as mean (SD) or frequency (%). Statistically significant difference: p<0.05 SD: standard deviation

	Group A (n=50)	Group B (n=50)	P-value
Demographic parameters			
Age (year)	55.7 (8.4)	56.2 (7.9)	
Sex (male/female)	14:36	20:30	
Height (cm)	170.3 (6.2)	169.8 (5.8)	
Weight (kg)	60.5 (9.3)	61.2 (8.7)	
Comorbidities			0.739
Diabetes	5 (10%)	3 (6%)	
Hypertension	3 (6%)	4 (8%)	
Hypothyroidism	2 (4%)	3 (6%)	
Diabetes and hypertension	3 (6%)	2 (4%)	
Nil	37 (74%)	38 (76%)	
Surgery			0.181
Breast	23 (46%)	14 (28%)	
Gastrointestinal	7 (14%)	12 (24%)	
Gynecological	3 (6%)	6 (12%)	
Head and neck	7 (14%)	5 (10%)	
Others	10 (20%)	13 (26%)	
Mouth opening (finger breath)			0.869
1	3 (6%)	2 (4%)	
2	8 (16%)	7 (14%)	
3	37 (74%)	40 (80%)	
4	2 (4%)	1 (2%)	
Mallampati grading			0.869
1	3 (6%)	2 (4%)	
2	8 (16%)	7 (14%)	
3	37 (74%)	40 (80%)	
4	2 (4%)	1 (2%)	
Neck flexion			0.012
Normal	44 (88%)	50 (100%)	
Restricted	6 (12%)	0 (0%)	
Neck extension			0.022
Normal	45 (90%)	50 (100%)	
Restricted	5 (10%)	0 (0%)	

Airway examination and pre-surgical findings

Key airway examination parameters, including mouth opening, Mallampati grading, neck flexion, and neck extension, were comparable between the telemedicine and face-to-face PAC groups. Table 1 shows that no significant differences were observed for any of these clinical indicators.

Furthermore, when the PAC findings were compared with the pre-surgical assessments, no clinically significant discrepancies were noted. This comparison confirmed that both modalities (telemedicine and face-to-face) provided equivalent accuracy in preoperative clinical evaluations, particularly with regard to airway management.

Physician satisfaction

Physician satisfaction, measured on a 1-10 scale, was comparable between the two groups, as shown in Table 2. Physicians rated the visualization of the patient, comfort during conversation, comprehension, and the thoroughness of information obtained similarly in both telemedicine and face-to-face PAC consultations. The mean physician satisfaction scores were 7.18 for telemedicine and 7.58 for face-to-face consultations (p=0.113), indicating no significant difference in satisfaction levels between the two methods.

**Table 2:** Physician satisfaction was assessed using a scale from 1 to 10, where 1 indicated strong disagreement and 10 indicated strong agreement Values are presented as means (SD). Statistically significant difference: p<0.05 CI, confidence interval; SD, standard deviation; PFT, pulmonary function test

	Group A (mean {SD})	Group B (mean {SD})	Absolute mean difference (Group A to Group B; 95% CI)	P-value
Visualization of the patient was good	7.44 (1.26)	7.94 (0.97)	-0.5 (-0.94 to -0.05)	0.029
Conversation with the patient was comfortable	7.10 (1.34)	8.08 (1.00)	-0.98 (-1.45 to -0.5)	0.0001
Comprehension of the conversation was good	6.78 (1.59)	7.80 (1.16)	-1.02 (-1.57 to -0.46)	0.0004
Thorough information could be obtained during the evaluation	7.12 (1.18)	7.80 (1.16)	-0.68 (-1.14 to -0.213)	0.0001
Thorough information pertaining to the oncological treatment of the patient could be obtained during evaluation	6.78 (1.26)	7.82 (1.24)	-1.04 (-1.53 to -0.54)	0.001
Thorough information pertaining to clinical examination of the patient could be obtained during evaluation	6.52 (1.51)	7.76 (1.33)	-1.24 (-1.80 to -0.67)	0.0001
Thorough information pertaining to blood investigations of the patient could be obtained during the evaluation	7.08 (1.39)	7.66 (1.34)	-0.58 (-1.12 to -0.03)	0.001
Thorough information pertaining to radiological investigations of the patient could be obtained during evaluation	7.00 (1.41)	7.38 (1.44)	-0.38 (-0.94 to 0.18)	0.1864
Thorough information pertaining to ECG, PFT, and PET scan could be obtained during evaluation	6.84 (1.36)	7.42 (1.51)	-0.58 (-1.15 to -0.08)	0.001
Information obtained through face-to-face/telemedicine consultation is more comprehensive compared to telemedicine/face-to-face consultation	6.86 (1.44)	7.66 (1.25)	-0.80 (-1.33 to -0.26)	0.001
Willing to use the same method of evaluation in the future for pre-anesthesia consultation	7.10 (1.09)	7.72 (1.16)	-0.62 (-1.06 to -0.17)	0.007
Overall, I am very satisfied with consultation	7.18 (1.24)	7.58 (1.26)	-0.4 (-0.89 to -0.09)	0.113

Patient satisfaction

Patient satisfaction was also high across both groups. Patients in the telemedicine group reported a similar level of satisfaction as those in the face-to-face group across various factors, including comfort during the consultation, comprehension of instructions, and the opportunity for a complete pre-anesthesia checkup. Patients particularly appreciated the convenience of avoiding hospital visits and the reduced waiting times associated with telemedicine consultations. As shown in Table 3, the mean patient satisfaction scores were 6.94 for telemedicine and 7.26 for face-to-face PAC (p=0.251), with no significant differences between the two groups.

**Table 3:** Patient satisfaction was assessed using a scale from 1 to 10, where 1 indicated strong disagreement and 10 indicated strong agreement Values are presented as means (SD). Statistically significant difference: p<0.05 CI, confidence interval; SD, standard deviation

	Group A (mean {SD})	Group B (mean {SD})	Absolute mean difference (Group A to Group B; 95% CI)	P-value
Conversation with the anesthesiologist was comfortable	7.18 (1.39)	7.26 (1.56)	-0.08 (-0.66 to 0.50)	0.7877
Could understand the directions and instructions provided to me by the anesthesiologist	7.16 (1.63)	7.14 (1.82)	0.02 (-0.66 to 0.70)	0.9541
The consultation gave me the opportunity for complete pre-anesthesia checkup before surgery	6.88 (1.40)	6.78 (1.91)	0.10 (-0.56 to 0.76)	0.005
The consultation gave the opportunity to avoid long waiting time in hospital premises	7.84 (1.59)	5.76 (2.44)	2.08 (1.26 to 2.89)	0.001
The consultation gave the opportunity to discuss the doubts related to anesthesia in comfortable environment	7.02 (1.49)	5.94 (2.21)	1.08 (0.33 to 1.82)	0.005
The consultation gave the opportunity to avoid travel to hospital, thus preventing extra expenditure	7.16 (1.29)	5.70 (2.54)	1.46 (0.65 to 2.26)	0.0005
Willing to be evaluated by the anesthesiologist using the same method again in future	7.36 (1.13)	6.58 (2.05)	0.78 (0.12 to 1.43)	0.0207
Overall, I am very satisfied with the consultation	6.94 (1.34)	7.26 (1.42)	-0.32 (-0.87 to 0.23)	0.2514

Duration of pre-anesthesia consultations

**Figure 2:**
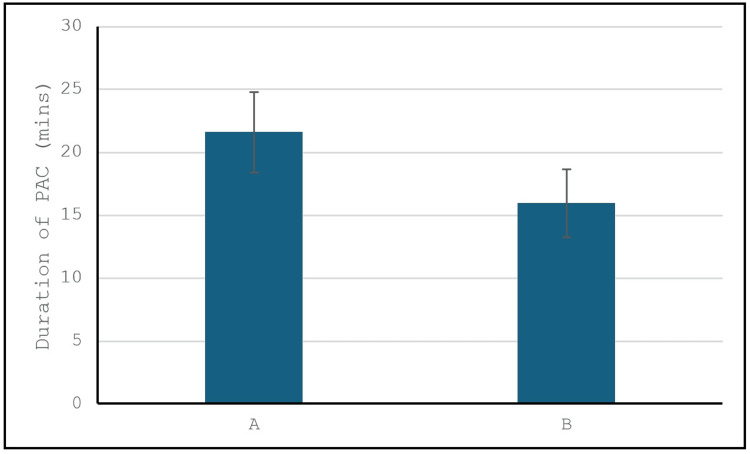
Comparison of the duration of PAC between Group A and Group B PAC: pre-anesthesia consultations

The average time required for the pre-anesthesia consultation was 21.58 minutes for telemedicine and 15.94 minutes for face-to-face consultations, as shown in Figure 2. Although telemedicine consultations took slightly longer, this difference did not affect the satisfaction scores and may be attributed to the technological interface.

Discrepancies between PAC and pre-surgical assessments

A secondary outcome of the study was the evaluation of any discrepancies between PAC findings and pre-surgical assessments. Importantly, no significant differences were found between the findings during the PAC (whether via telemedicine or in-person) and the pre-surgical assessments conducted immediately before surgery. This confirmed the clinical reliability of telemedicine PAC in accurately assessing preoperative parameters, including airway examination.

## Discussion

Telemedicine, the use of information and communication technologies to deliver healthcare services, offers several benefits, including enhanced access to care, reduced travel time and costs, and improved communication between healthcare providers and patients [12]. However, it also presents challenges such as technological limitations, medicolegal concerns, and the need for adequate training and infrastructure [13]. These challenges are especially relevant in developing countries such as India, where the necessary technology for advanced teleconsultations is still emerging and legal frameworks for telemedicine are yet to be fully established.

In this prospective, randomized non-inferiority study, conducted at a tertiary cancer care center, we aimed to evaluate the feasibility and clinical effectiveness of telemedicine as an alternative to traditional face-to-face pre-anesthesia consultations (PAC). Rather than aiming to demonstrate superiority, our goal was to confirm that telemedicine PAC is non-inferior to in-person consultations in terms of patient and physician satisfaction, as well as clinical accuracy. Our primary outcomes focused on satisfaction, while secondary outcomes evaluated the clinical accuracy of airway examination and any discrepancies between pre-anesthesia consultations and pre-surgical assessments.

Our results demonstrated that telemedicine PAC was non-inferior to face-to-face PAC in both physician and patient satisfaction. Physicians reported similar levels of satisfaction across factors such as the quality of visualization, conversation comfort, and the thoroughness of information obtained [14,15]. Importantly, no significant difficulties were reported regarding the quality of the audio/visual technology or the ability to perform clinical evaluations via telemedicine. These findings are consistent with previous studies that have shown telemedicine to be an effective tool for pre-anesthesia evaluations, providing physicians with the necessary clinical data to make informed preoperative decisions [14,15].

The comparison of airway examination parameters between the two groups further supports the clinical validity of telemedicine. Parameters such as mouth opening, Mallampati grading, neck flexion, and neck extension were comparable between the telemedicine and face-to-face groups, and no significant discrepancies were observed between the initial PAC findings and the pre-surgical assessments. This indicates that telemedicine can reliably assess critical clinical indicators relevant to anesthesia management, aligning with other studies that have demonstrated the effectiveness of telemedicine in maintaining clinical accuracy [14,15].

Patient satisfaction was also high across both groups, with telemedicine participants appreciating the convenience of avoiding travel to the hospital and reduced waiting times. Patients in the telemedicine group reported a similar level of satisfaction with factors such as conversation comfort, comprehension of instructions, and the opportunity for a complete pre-anesthesia checkup. This is in line with previous research highlighting the convenience and resource optimization offered by telemedicine [16].

However, some challenges remain. As telemedicine continues to evolve, patient concerns about depersonalization and unfamiliarity with virtual consultations need to be addressed. For some patients, interacting with healthcare providers through a screen may feel less reassuring than in-person consultations [17]. This could explain why patients receiving face-to-face consultations were found to be slightly better at anticipating their anesthetic experience on the day of surgery, as they had more direct interactions with their anesthesiologist. Future research could explore ways to enhance the interpersonal aspects of telemedicine consultations to mitigate these concerns.

Our findings align with previous research by Applegate et al. [14] and Zetterman et al. [15], which reported high patient and provider satisfaction with telemedicine pre-anesthesia evaluations, emphasizing its equivalence to traditional methods. Furthermore, studies by Kamdar et al. [10] and Lozada et al. [11] also demonstrated the efficiency and convenience of telephonic and electronic preoperative assessments, contributing to improved patient experiences and resource optimization [18].

Limitations and challenges

Despite promising results, several limitations must be acknowledged. Technical issues, including unreliable internet and limited device access, remain barriers to broader adoption. Medicolegal considerations and clear regulatory frameworks are essential for the safe use of telemedicine [13]. Another important factor is the evolving nature of telemedicine during and after the COVID-19 pandemic. The telemedicine PAC in this study was initiated in response to pandemic-related restrictions, and patient and physician preferences may have changed as these restrictions eased.

The inability to perform physical examinations such as auscultation, palpation, or cardiac assessments limits diagnostic accuracy in virtual consultations. Spine assessments for regional anesthesia also pose challenges due to the lack of palpation.

Language barriers were encountered in three cases, where relatives assisted with communication, highlighting the need for interpreters or multilingual support. The unblinded nature of the study may also introduce bias in subjective satisfaction scores, despite independent assessments.

Lastly, the generalizability of findings is limited by the specific patient population (oncology patients) and setting (tertiary cancer care center). Complex cases requiring specialist consultations (e.g., nephrology or cardiology) further reduce the feasibility of teleconsultations. Broader studies across diverse patient groups and healthcare settings are needed to validate the role of telemedicine in pre-anesthesia consultations.

## Conclusions

This study establishes telemedicine as a non-inferior alternative to face-to-face pre-anesthesia consultations in a tertiary cancer care setting. It achieves comparable patient and physician satisfaction while maintaining clinical accuracy in key areas such as airway assessment. Patients valued its convenience, including reduced travel and waiting times.

However, telemedicine facilitates only a partial assessment, as physical examinations requiring palpation or auscultation are not possible. Complex cases requiring detailed evaluations or specialist input may necessitate hybrid models or in-person consultations. Future studies should explore integrating advanced diagnostic technologies, cost-effectiveness, and broader patient populations to validate telemedicine’s role in diverse settings. Despite its limitations, telemedicine offers significant potential to enhance accessibility and optimize resources in high-demand care environments.
